# Illuminating the prefrontal neural correlates of action sequence disassembling in response–response binding

**DOI:** 10.1038/s41598-021-02247-6

**Published:** 2021-11-24

**Authors:** Christoph F. Geissler, Christian Frings, Birte Moeller

**Affiliations:** Department of Cognitive Psychology, University of Trier, 54286 Trier, Germany

**Keywords:** Human behaviour, Cognitive neuroscience

## Abstract

Execution of two independent actions in quick succession results in transient binding of these two actions. Subsequent repetition of any of these actions automatically retrieves the other. This process is probably fundamental for developing complex action sequences. However, rigid bindings between two actions are not always adaptive. Sometimes, it is necessary to repeat only one of the two previously executed actions. In such situations, stored action sequences must be disassembled, for the sake of flexibility. Exact mechanisms that allow for such an active unbinding of actions remain largely unknown, but it stands to reason, that some form of prefrontal executive control is necessary. Building on prior neuronal research that explored other forms of binding (e.g. between distractors and responses and abstract representations and responses), we explored middle and superior frontal correlates of -response binding in a sequential classification task with functional near-infrared spectroscopy. We found that anterior dorsolateral prefrontal cortex activity varied as a function of response–repetition condition. Activity in the right anterior dorsolateral prefrontal cortex correlated with changes in reaction times due to response–response binding. Our results indicate that the right anterior dorsolateral prefrontal cortex dismantles bindings between consecutive actions, whenever such bindings interfere with current action goals.

## Introduction

A central question in action-control research is, how stimulus features are integrated with actions features to derive working action plans^1^. A theoretical framework for such perception–action integration is provided by the theory of event coding (TEC^2,3^). According to TEC, whenever an action is executed, this action, as well as perceptual features of the surrounding are integrated into an event-file, which can be retrieved at a later point in time. The more recent Binding and Retrieval in Action Control (BRAC) framework^4^ extends the assumptions of TEC and emphasizes that event-file integration and retrieval are distinct processes that can be independently modulated by several bottom up and top down influences. Both TEC and BRAC assume that event-files are created by binding stimulus and action features together and that a later repetition of one of the features retrieves the others, which in turn can influence subsequent actions. A full repetition of the contents of an event-file leads to facilitation of subsequent actions, while a partial repetition of the event-file contents typically leads to interference with the subsequent action. Together, this facilitative and interference effects of event-file retrieval constitute the so called binding effect.

In the laboratory, the influence of binding on action execution is usually measured with a sequential task design. In the first event (the prime), stimulus and response features of the event are integrated and repetition of any of these features at the second event (the probe) can retrieve the previous episode thereby influencing probe responding. Binding effects occur not only due to integration and retrieval between target stimuli and responses^5^, but also due to integration between distractor stimuli and responses^6^ and effect stimuli and responses^7^. Even tasks^8^ and control states^9^ have been shown to be integrated with and retrieve responses. Besides the large data base providing evidence for stimulus–response binding effects, there is recent evidence also for integration and retrieval of individually planned and executed actions^10^, possibly providing the basis for representations of complex action sequences.

For the present purpose, it is important to note that the temporal stability of binding effects depends on the type of binding. While response-irrelevant distractor stimuli are reliably integrated with responses^6,11,12^, distractor–response binding effects reliably decay within a couple of seconds after integration^13–16^. Bindings between target stimuli and responses can be measured 4 s after integration^17^, but seem to fully decay after about 5 s^18^. Finally, bindings between individual responses do not seem to decay measurably within the first 6 s after integration^19^ and thus may be optimally suited to neurophysiologic studies of binding mechanisms—however, to the best of our knowledge, not a single study using this kind of binding task has been reported so far.

### Neural correlates of binding

Imaging research has the potential to further deepen our understanding of the distinct processes involved in binding. However, it is somewhat hampered by the transitory nature of event-files discussed above. The reason for this being that the hemodynamic response, which is the basis for imaging procedures like functional magnetic resonance imaging (fMRI) and functional near-infrared spectroscopy (fNIRS), has a linear relationship to neural activity only at stimulus onset asynchronies (SOAs) over 2 s^20,21^. Additionally, it is highly advised to implement variable inter trial intervals (usually somewhere between 2 to 6 s) to increase the power of rapid event related fMRI and fNIRS designs^21^. While target-response binding (but not distractor–response binding) can still be detected after 4 s, the effects are substantially smaller than after 1 or 2 s^17^. After 6 s target-response binding effects can be expected to have vanished completely^18^. As a consequence, imaging methods that rely on the hemodynamic response cannot clearly distinguish between integration and retrieval related processes in target-response or distractor–response binding, because the length of SOAs necessary to distinguish between prime and probe related activity might compromise binding effects. Consequently, much of the prior research into the neural correlates of binding has instead employed EEG, which does not suffer from the same problems of low temporal resolution, but has the drawback of providing only limited spatial resolution.

Recently, several EEG studies have employed different stimulus response binding paradigms, to investigate the electrophysiological correlates of binding. These studies primarily found binding-related activation in parietal regions in the form of greater P300^22–24^ and N450^25^ amplitudes in partial repetition trials. Additionally, Opitz et al.^25^ found binding related activity in the form of greater N450 amplitudes in partial repetition trials in the middle frontal gyrus (MFG). Another study by Pastötter et al.^16^ analyzed oscillatory brain activities related to the integration and disintegration of event-files in distractor response binding. Unsurprisingly, mean behavioral distractor–response binding effects were significant when measured 500 ms after integration, but not when measured 2000 ms after integration. Interestingly however, EEG results revealed a positive correlation between individual distractor–response binding effects in the 2000 ms response–stimulus-interval condition and post-movement occipital beta synchronization after both prime and probe responses. This is a first indication that post-movement beta synchronization is a marker of event-file disintegration, with more stable synchronization if bindings last somewhat longer.

Despite its problem of low temporal resolution, there have been some attempts to provide a more detailed mapping of neural regions involved in binding processes with fMRI. Kühn et al.^26^ employed a detection/identification task with face and house stimuli and found that partial repetition of a previous stimulus–response episode led to inhibition of areas related to the components that were not repeated (i.e. the right or left motor cortex for non-repeated left or right responses respectively, or the fusiform face area or parahippocampal place area, for non-repeated face or house stimuli). Kühn et al.^26^ suggest, that this pattern of results indicates that the non-repeated components were automatically retrieved and had to be inhibited to reduce interference with current trial execution. Another study by Pollmann et al.^27^, though not directly investigating binding, examined sequential effects in a selection of singleton task and found activity in the parieto-occipital fissure that suggests binding between the discriminating stimulus feature dimension and the response.

Additionally, there have been imaging studies to phenomena akin to binding, for example long-lag repetition priming (LLRP). LLRP is usually examined in some form of classification task. Thus, for example, a decision (yes or no) must be made for a series of items, in regards to whether each item is larger (or smaller) than a reference item^28–33^. LLRP produces similar behavioral results to those found in binding paradigms. Thus, if the response to a previously presented item has to be repeated in a later trial, RTs and error rates decline^29–31,33–36^. On the other hand, if the response made to an item previously changes in a later trial, RTs and error rates increase compared to newly presented items^29,35,36^. Contrary to binding effects, LLRP effects last for time intervals of several minutes^28–31,34,35^. Consequently, LLRP arguably does not reflect pure binding, but rather a form of more stable contingency learning. Nonetheless, due to the similarities of both effect types, insights from LLRP studies might point to some of the neural structures implicated in binding. For example, Dobbins et al.^34^ found that neural activity in prefrontal, parietal, occipito-temporal and fusiform regions declined when repeatedly executing the same response to a specific stimulus in a classification task. An effect that was partially abolished in the prefrontal cortex and fusiform gyrus when the response to an item changed in a later trial. Horner and Henson^29,31^ and Race et al.^36,37^ expanded on this finding by demonstrating that similar congruency effects in the neural response prevailed when the stimulus material or the underlying classification task changed. Importantly, Horner and Henson^31^ could also show, that a change in response to an item resulted in a neural interference effect that was reflected in higher activity in the right inferior frontal gyrus, the right MFG and the right insula in trials where a different reaction to a previously presented item had to be made, compared to trials with novel items.

### The present study

**Figure 1:**
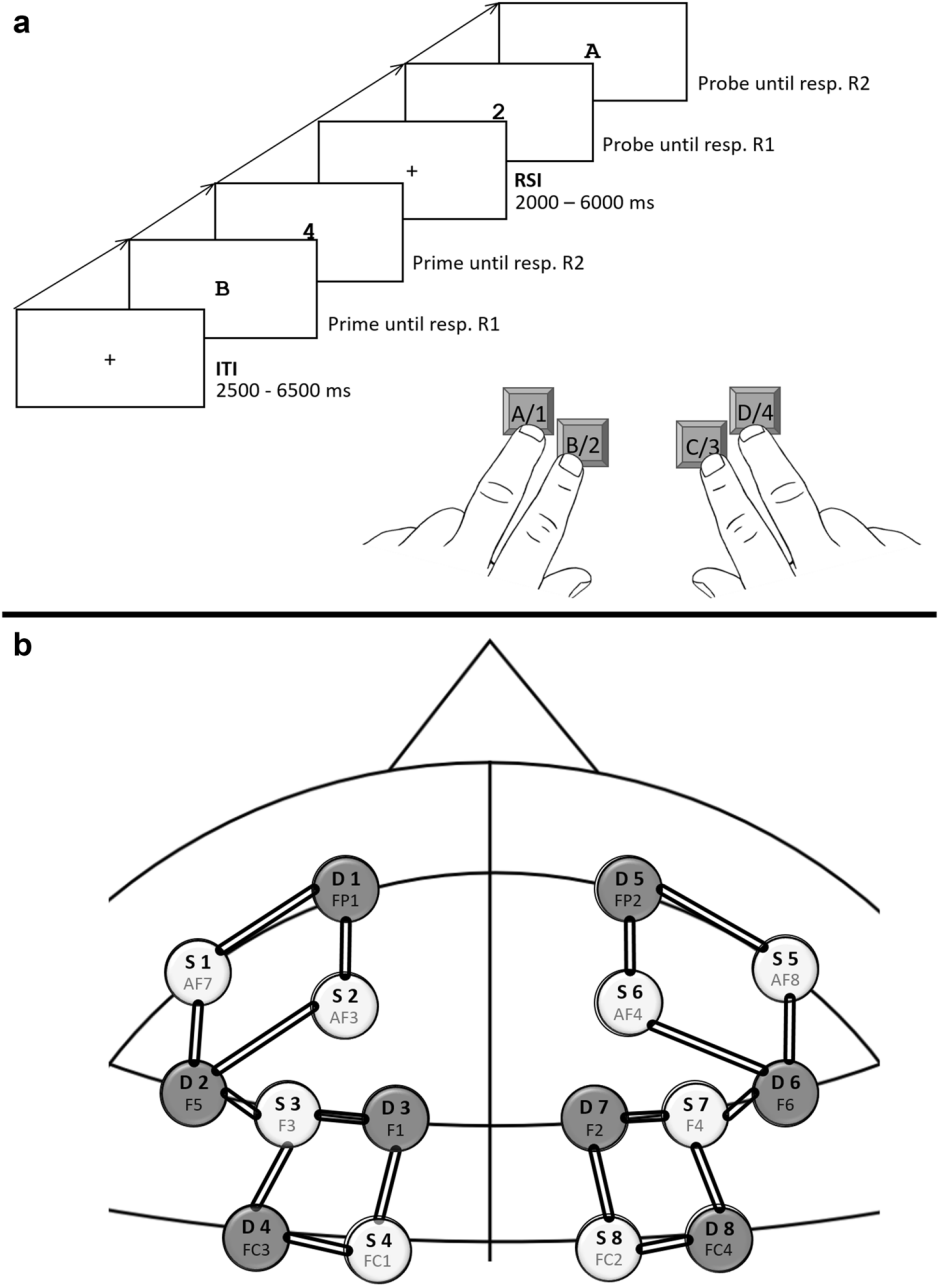
(**a**) Trial procedure. Participants’ task was to press the key corresponding to individually presented digits and letters (A/1, B/2, C/3, or D/4). The depicted trial is an example for a response R1 repetition and response R2 change trial. Note: White is depicted in black and black is depicted in white. (**b**) fNIRS Montage. AF3, AF4, AF7, AF8, F3, F4, FC1 and FC2 were chosen as source positions. FP1, FP2, F1, F2, F5, F6, FC3 and FC4 were chosen as detector positions. These positions where chosen to allow for an optimal coverage of the MFG and resulted in eighteen channels.

Building on the results of several studies that found binding-related changes in prefrontal activation^25,31,34^, we examined MFG and superior frontal gyrus (SFG) activity during a response–response binding (RRB) task with near-infrared spectroscopy (fNIRS). The task consisted of a discrimination task in a prime-probe design. During each prime and probe participants responded to two consecutive targets by executing two key presses in quick succession (see Fig. 1a). Probe responses could either be a full repetition of the prime responses (i.e., both responses were the same in prime and probe), or a partial repetition of the prime responses (i.e. only exactly the first or the second probe response was repeated from the prime response), or they could fully change from prime responses (i.e., both probe responses were different from the prime responses). We chose RRB as binding paradigm because, as outlined above, it produces comparatively large and temporally stable binding effects, which made it possible to separate the prime and probe by several seconds. This, in theory, should allow us to clearly separate probe related activity from any lingering prime related neural activity and thus to capture neural processes specifically related to event-file retrieval. fNIRS was chosen as an imaging method, because it allows for disturbance-free and inexpensive measurements of cortical brain activity (see Fig. 1b for optode mounting). It utilizes near-infrared light to simultaneously measure changes in oxygenated hemoglobin ([oxyHB]) and deoxygenated hemoglobin ([deoxyHB]). Thus, either a rise in [oxyHB] or a decline in [deoxyHB] can signal a rise in neural activity. Both [oxyHB] and [deoxyHB] also show substantial correlations with the blood-oxygen-level-dependent (BOLD) signal of the fMRI^38,39^.

Altogether, we had three hypotheses regarding the results of our experiment. First, we expected to replicate the behavioral results of previous RRB experiments. Thus, we anticipated a significant binding effect in both response times (RTs) and error rates (ERs). Second, we expected to find a neural binding effect. Thus we anticipated the modulation of MFG activity as function of prime-probe relation analogue to the behavioral effects, i.e. lower activation due to facilitation in full repetition trials and higher activation due to interference in partial repetition trials. Third, if the DLPFC is functionally involved in event-file retrieval, we expected that the neural and the behavioral binding effects would be correlated.

## Results

The average response accuracy (AC) was 97% (SD = 2%), the average response time (RT) for correct reactions was 675 ms (SD = 83 ms). All reported RT analyses refer only to correct responses.

### Behavioral data

If the two prime responses R1 and R2 were integrated, repeating prime response R1 as probe R1 may trigger retrieval of prime R2, which would then influence probe R2 performance. Therefore, performance in probe R2 was the dependent variable of interest. Only trials with correct responses both in prime and probe were considered for the analysis of probe R2 response times (RTs). The rate for at least one error in the prime responses was 7.3%. Probe error rates were 3.0% for R1, and 4.6% for R2 (only including trials without errors in the previous responses). RTs that were more than 1.5 interquartile ranges above the third quartile of the participant’s RT distribution^40^ and RTs, below 200 ms were excluded from the analyses. Due to these constraints, 16.8% of the trials were excluded from the RT analyses. See Table 1 for condition-wise behavioral results.

**Table 1:** Mean response times (in ms) and mean error rates (in percent) for probe R2 responses, as a function of probe R1 and R2 relation to prime R1 and R2.

	R2^b^ repetition		R2 change	
	Response times	Error rates	Response times	Error rates
R1^a^ change	529	5.6	520	3.6
R1 repetition	501	2.3	538	6.8
Priming Effect	28	3.3	− 18	− 3.2

^a^Reaction 1.

^b^Reaction 2.

**Figure 2:**
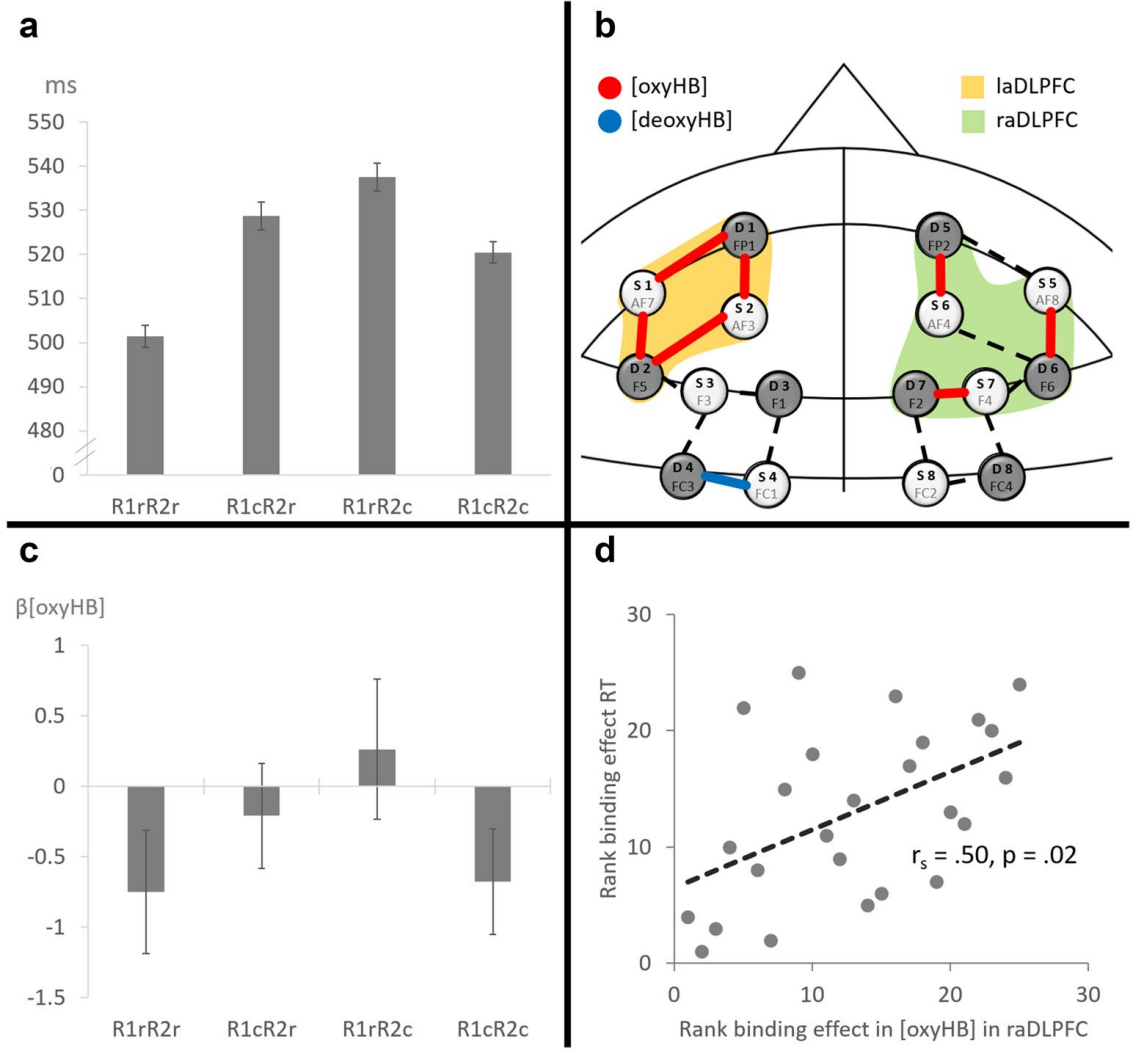
(**a**) Reaction times in the different response relation conditions. Error bars indicate within-subject design confidence intervals as suggested by Cousineau^41^. (**b**) Binding effect in neural activation and ROIs. Depicts the channels that showed changes in activation significant from 0 for the binding contrast [(R1cR2r–R1rR2r)–(R1cR2c–R1rR2c)] reflected by [oxyHB] or [deoxyHB]. The yellow and green areas reflect the laDLPFC and raDLPFC ROIs respectively that were built post hoc for correlation analysis. (**c**) Changes in oxyHB in the different response relation conditions in raDLPFC. Bars depicts the mean first level betas over all participants. Error bars indicate within-subject design confidence intervals as suggested by Cousineau^41^ for the contrast between conditions and provide no information about blood flow increases or decreases from baseline. (**d**) Rank correlation between neural binding effect in raDLPFC ROI and behavioral binding effect in reaction times.

In a 2 (R1 relation: repetition vs. change) × 2 (R2 relation: repetition vs. change) MANOVA on probe response R2 RTs with Pillai’s trace as the criterion, the main effect of R2 relation reached significance, *F*(1,24) = 11.48, *p* = 0.002, *η*_p_^2^ = 0.32, while the main effect of R1 relation did not, *F*(1,24) = 3.36, *p* = 0.079, *η*_p_^2^ = 0.12. Importantly, the interaction of R1 and R2 relation was significant as well, *F*(1,24) = 54.91, *p* < 0.001, *η*_p_^2^ = 0.70, indicating a significant response–response binding effect. Follow up analyses specified that repeating R1 from the prime facilitated R2 execution (by 27 ms) only if R2 also repeated from prime to probe, *t*(24) = 6.54, *p* < 0.001, *d* = 1.31. If R2 changed from prime to probe R1 repetition impaired R2 execution (by 17 ms), *t*(24) = 4.33, *p* < 0.001, *d* = 0.87, adding up to a mean response–response binding effect of 44 ms (SD = 30 ms, see Fig. 2a for reaction times in the different response relation conditions). In the same analyses on error rates, only the interaction of R1 relation and R2 relation was significant, *F*(1,24) = 22.71, *p* < 0.001, *η*_p_^2^ = 0.49. Repeating R1 from the prime facilitated R2 execution (3.3% fewer errors) only if R2 also repeated from prime to probe, *t*(24) = 4.0, *p* = 0.001, *d* = 0.80. If R2 changed from prime to probe R1 repetition impaired R2 execution (3.3% more errors), *t*(24) = 3.29, *p* = 0.003, *d* = 0.66, resulting in a mean binding effect of 6.6% errors.

### Neural data

On a neural level, the binding contrast [(R1cR2r–R1rR2r)–(R1cR2c–R1rR2c)], testing the interaction of R1 and R2 relation produced 8 significant effects. Activated regions included the lMFG (4 significant contrasts), the rMFG (2 significant contrasts), the left SFG (1 significant contrast) and the right SFG (1 significant contrast). The majority of the effects were found in [oxyHB] (7 significant contrasts) but there was also one effects in [deoxyHB]. All significant [oxyHB] contrasts produced positive effects, while the significant [deoxyHB] contrast produced a negative effect. No channel showed significant changes in blood concentrations related to prime-presentation (all p > 0.05). See Table 2 for functional hemodynamic results. See Fig. 2b for localization of the functional hemodynamic effects.

**Table 2:** Hemodynamic results depicting all significant binding contrasts in oxygenated and deoxygenated hemoglobin.

Brainregion	Channel	MNI X, Y, Z^a^	HB-type	β (SE)^b^	t	p
lMFG	AF7-FP1	− 33, 59, − 2	Oxygenated	1.11 (0.34)	3.28	0.01
	AF7-F5	− 47, 46, 6	Oxygenated	0.80 (0.025)	3.26	0.01
	AF3-F5	− 39, 50, 17	Oxygenated	0.92 (0.34)	2.66	0.04
	FC1-FC3	− 38, 12, 55	Deoxygenated	− 0.58 (0.22)	− 2.68	0.04
rMFG	AF8-F6	48, 46, 5	oxygenated	0.58 (0.21)	2.74	0.04
	F4-F2	30, 40, 41	Oxygenated	1.73 (0.21)	8.41	< 0.001
lSFG	AF3-FP1	− 24, 63, 9	Oxygenated	0.93 (0.25)	3.66	< 0.01
rSFG	AF4-FP2	25, 63, 9	Oxygenated	1.46 (0.25)	5.81	< 0.001

^a^MNI-coordinates reflect the respective central voxel of the found effects.

^b^All contrast β are depicted with standard errors. All p reflect pFDR corrected significances.

To test whether the neural and behavioral binding effects were related, two ROIs were built. One ROI encompassing the four channels that produced significant contrasts in the lMFG (AF7-FP1, AF7-F5, AF3-FP1, AF3-F5), termed left anterior DLPFC (laDLPFC) and one ROI encompassing the three channels that produced significant contrasts in the right hemisphere as well as two additional channels that lay in the middle of the activated areas (AF8-F6, AF4-FP2, AF4-F6, F4-F6, F4-F2), termed right anterior DLPFC (raDLPFC). See Fig. 2b for localization of both ROIs. See Fig. 2c for changes in oxyHB in the different response relation conditions in the raDLPFC ROI. Binding related activation in raDLPFC correlated significantly with the behavioral binding effect in RTs (r_s_ = 0.50, p = 0.02; see Fig. 2d), while binding related activation in the laDLPFC showed no such correlation (r_s_ = 0.06, p = 0.77).

## Discussion

In this study, we investigated the frontal hemodynamics of response–response binding in a prime-probe design^10^ with fNIRS. On a behavioral level, we found significant binding effects in both reaction times and error rates. Our behavioral findings mirror the results of earlier studies employing this paradigm^10,19,42,43^ and were expected with the theoretical predictions of both the TEC^2^ and the BRAC framework^4^ in mind.

Importantly, we also found significant binding effects in neural activity. That is, probe activation was relatively higher in probe trials in which we would expect retrieved event-files to be incompatible with current action goals than in trials in which we would expect retrieved event-files to be compatible with current action goals. These effects were present mostly in [oxyHB] and focused on several channels in the bilateral anterior MFG. Moreover, neural binding effects in the right anterior MFG/SFG were positively correlated with behavioral binding effects, indicating that participants who showed the highest evidence for neural binding effects also produced larger behavioral binding effects. Hence, we were able to pinpoint an area that seems to be integral to processing in a central stage of action control—namely response retrieval.

Intriguingly, only the neural binding effect in the right anterior MFG/ SFG activity but not the neural binding effect in left anterior MFG activity was directly related to the behavioral binding effects. This was indicated by a significant positive correlation between binding related activity in a raDLPFC ROI which encompassed channels in the general area where the neural binding effect was found in the right hemisphere and the binding effect in reaction times, and an absence of a corresponding correlation between the activation of a laDLPFC ROI encompassing channels that showed a significant binding effect in the left hemisphere and behavioral binding. Such a somewhat closer relationship between right-sided prefrontal activity and behavioral binding effects is reminiscent of earlier findings that specifically related right MFG activity to the binding of distractors and responses^25^ and abstract representations of stimuli and responses^31^ respectively.

The relative temporal stability of response–response bindings^19^ enabled us to employ varying SOAs of 2 to 6 s, which allowed us to clearly distinguish probe and prime related neural processes, without risking a dilution of the binding effect. Accordingly, probe-related activity changes in our design can most likely be ascribed to neural processing specifically related to event-file retrieval. However, the changes we report do not seem to represent the process of retrieving event-files itself, which would be reflected in significantly lower activity in full change (where nothing is repeated that might start retrieval) compared to all other probe types (where always at least one response is repeated and might start retrieval). Rather, the significant binding contrast in probe activity points to distinct processes related to the compatibility of a retrieved event-file with current task demands. Specifically, incompatibility of a retrieved event file with a current event-file seems to lead to an increase in activity.

The occurrence of such an effect in a prefrontal area suggests the involvement of some executive control process in the processing of retrieved event-files. Beyond a close relationship of prefrontal structures in executive control in general^44–46^, this is indicated by prior binding research, which has explicitly contemplated the involvement of prefrontal executive processes in event-file management^26,47^. Only recently, it has been argued, that the rMFG might act as a monitor of conflict between existing bindings and current task objectives^25^. However, given our finding of a significant correlation between a neural binding effect in this area and a behavioral binding effect in reaction times, we propose that the rMFG/SFG more directly transforms event-file contents to reach an alignment with current task goals.

This is in line with the proposal that the prefrontal cortex achieves the resolution of conflict between an existing event-file and task demands by actively unbinding the event-files contents^47^. We argue that such an active decomposition of an event-file (contrary to more passive event-file decay which does not seem to involve prefrontal structures^16^) might be accomplished by the rDLPFC, an important functional network spanning both rMFG and rSFG. Although beyond the scope of prior binding literature, this notion fits well with working memory research that has related the DLPFC to active memory updating in general and specifically the rDLPFC to updating of both simple^48^ and complex action plans^49^.

In conclusion, we used fNIRS to examine the neuro hemodynamics of response–response binding. In accordance to previous binding research, we found significant binding effects in both reaction times and accuracies and MFG activation. Additionally, the neural binding effect in the right MFG and SFG was significantly correlated to the behavioral binding effect in reaction times. We argue that the right DLPFC disassembles event-files containing previously built action plans that interfere with current task objectives.

## Methods

### Participants

Twenty-five subjects were included in the study (18 female; mean age = 21.92, SD = 2.66), all of whom participated either as part of the curriculum for the psychology bachelor at Trier University or as a friendly turn and interest in fNIRS methodology. G*Power (v3.1.9.7^50^) sensitivity analysis for one-sample t-test indicated a required medium effect size of d = 0.58 (two-tailed) when alpha was set to 0.05 and power to 0.80. All participants stated normal or corrected-to-normal vision; no participant stated any history of neurological disease. Participants gave written informed consent to participation as well as publication of anonymized data before examination. The study was conducted in accordance with the Declaration of Helsinki. Furthermore, the local ethical review committee at the University of Trier evaluated and approved the study (Decision regarding motion 72/2018, 11.12.2018).

### Design

The design included two within subject factors; namely Response 1 relation (repetition vs. change from prime to probe) and Response 2 relation (repetition vs. change from prime to probe).

### Material

The experiment was programmed using E-prime 3.0. Instructions and stimuli were presented in white on black background on a standard TFT screen. Stimuli were the digits 1, 2, 3, and 4 and the letters A, B, C, and D. All digits and letters subtended a horizontal visual angle of 0.4° to 0.6°, and vertical visual angle of 0.5°. Participants had a viewing distance of approximately 60 cm and responded by pressing one of four keys on the computer keyboard.

### Procedure

The procedure was adapted from previous studies^19,42^. Participants were tested individually in a dimly lit room. Instructions were given both verbally and via screen. Participants were instructed to place middle and index fingers of both hands on the keys S, C, M, and L (marked with A/1, B/2, C/3, and D/4) of a standard QWERTZ-keyboard. Their task was always to press the key corresponding to individually presented digits and letters. All stimuli were presented in the middle of the screen. Each trial started with the presentation of the first prime digit or letter, indicating prime R1 response until the participant pressed one of the four response keys. Then the second prime stimulus appeared indicating prime R2 response until a response was detected, which was followed by a blank response stimulus interval that varied randomly between 2000 and 6000 ms. Then the first probe stimulus appeared, indicating probe R1 until a response was detected which was again followed by the second probe stimulus, indicating probe R2 until response detection. Inter trial intervals varied randomly between 2500 and 6500 ms (see Fig. 1a for trial procedure). Every 64 trials participants were prompted to take a short break and got feedback on their performance in the last block, after which they resumed the task in their own time. An error message was presented for 1500 ms immediately following any erroneous response during practice. No error messages were presented during the experimental block.

The two factors relation of R1 response between prime and probe (repetition vs. change) and relation of R2 response (repetition vs. change) were varied orthogonally, while stimuli did not repeat between prime and probe. In R1 repetition trials (R1r), the presented stimuli required the same response as prime R1 response and probe R1 response. In R1 change trials (R1c), the presented stimuli required different responses as prime R1 response and probe R1 response. In R2 repetition trials (R2r), the presented stimuli required identical responses as prime R2 response and probe R2 response. In R2 change trials (R2c), the presented stimuli required different responses as prime R2 response and probe R2 response. Overall there were 256 experimental trials (64 of each of the four conditions R1rR2r, R1rR2c, R1cR2r, R1cR2c). The practice phase included 16 trials (a subset of the trials in the experimental block).

### fNIRS measurment

Hemodynamic changes were recorded with an eight source, eight detector, portable, time-multiplexed, two wavelengths NIRSport™ (NIRx Medical Technologies LLC, USA) fNIRS device. Optodes were fixed in a standard, 10–10 NIRScaps™ (NIRx Medical Technologies LLC, USA). The fNIRS Optodes' Location Decider (fOLDv2.2^51^) was used to determine optode placement. fOLD is a Matlab (MathsWorks, USA) based toolbox which computes optimal optode placement in the 10-10 system in regards of covering specific brain areas. AF3, AF4, AF7, AF8, F3, F4, FC1 and FC2 were computed as source positions and FP1, FP2, F1, F2, F5, F6, FC3 and FC4 were computed as detector positions for optimal coverage of the MFG. This resulted in eighteen different channels each recording the MFG with a specificity of 22.44% or greater (Fig. 1b depicts the fNIRS Montage). Signals were recorded with a frequency of 7.81 Hz and digitalized with NIRStar™ (NIRx Medical Technologies LLC, USA) recording software.

### fNIRS data preprocessing and analysis

Raw data was preprocessed and subsequently analyzed with NIRS Brain AnalyzIR Toolbox^52^. First, raw voltage data was transformed into light-intensity data, which was then used to calculate the relative concentration of oxygenated and deoxygenated hemoglobin via Beer–Lambert–Law^53^. Finally, to remove low-frequency characteristics and outliers, a wavelet-filter^54^ was applied. The preprocessed data was subsequently entered into a two-level GLM. The first level analysis included eight predictors and was conducted for each subject separately. Four predictors (one for each response–response binding condition) coded probe-related activity in error free experimental trials. An additional predictor coded prime activity in error free experimental trials. To account for neural activity related to erroneous trials^55,56^, three predictors were included, one coding probe activity in erroneous trials, one coding prime-related activity in trials with prime error and one coding prime related activity in trials with probe error. The latter three predictors were excluded from second level analysis. GLM predictors were generated by convolving each event with the canonical hemodynamic response function (HRF). To adapt modeling for individual differences in onset and dispersion of HRF we included the first and second temporal derivative of each prediction term. We corrected for serially auto-correlated errors as well as artifacts induced by systemic physiology and motion with an algorithm encompassing both prewhitening and robust regression (AR-IRLS^57^). For the second level analysis, the beta values obtained for each experimental condition for each subject were entered into a weighted mixed effects model estimating a fixed intercept for each experimental condition and a random intercept for each subject to best fit the overall data. To determine the neural binding effect, the resulting betas for each condition were entered into the contrast for the interaction of Response 1 and Response 2 relation [(R1cR2r–R1rR2r)–(R1cR2c–R1rR2c)] which was subsequently tested against zero via t-test. To test the relation between the behavioral and neural binding effect, regions of interest (ROIs) encompassing each broader area of neural activation were built as described in Santosa et al.^52^. Again, only predictors coding probe activation in error-free trials were included into the analysis. Subject-level betas for each condition for each ROI were entered into the binding contrast which again was tested against zero via t-test. The resulting t-values for each subject were then correlated with the behavioral binding effect in reaction times using rank correlation^58^. To account for alpha inflation due to multiple comparisons all p-values were corrected applying positive false discovery rate (FDR^59^). Only contrasts and correlations that yielded corrected *p* < 0.05 were regarded as statistically significant.

## Data Availability

Our data is publicly available via PsychArchives (http://dx.doi.org/10.23668/psycharchives.5215).

## Code availability

Our code for statistical analysis is publicly available via PsychArchives (http://dx.doi.org/10.23668/psycharchives.5214).
